# A Telemedicine System Intervention for Patients With Type 1 Diabetes: Pilot Feasibility Crossover Intervention Study

**DOI:** 10.2196/35064

**Published:** 2023-04-28

**Authors:** Martina Vlasakova, Jan Muzik, Anna Holubová, Dominik Fiala, Eirik Arsand, Jana Urbanová, Denisa Janíčková Žďárská, Marek Brabec, Jan Brož

**Affiliations:** 1 First Faculty of Medicine Charles University Prague Czech Republic; 2 Department of Information and Communication Technologies in Medicine Faculty of Biomedical Engineering Czech Technical University in Prague Praha Czech Republic; 3 University of Tromsø – The Arctic University of Norway Tromsø Norway; 4 Norwegian Centre for E-health Research Tromsø Norway; 5 Department of Internal Medicine, Faculty Hospital Kralovske Vinohrady Center for Research in Diabetes, Metabolism and Nutrition Charles University Third Faculty of Medicine Prague Czech Republic; 6 Department of Internal Medicine Second Medical Faculty Charles University and Motol University Hospital Prague Czech Republic; 7 Institute of Computer Science Academy of Science of the Czech Republic Prague Czech Republic

**Keywords:** diabetes mellitus, diabetes, telemedicine, telemedicine system, mobile health, mHealth, telemonitoring, quality of life, telehealth, compensation, evaluation, intervention, feasibility

## Abstract

**Background:**

Today’s diabetes-oriented telemedicine systems can gather and analyze many parameters like blood glucose levels, carbohydrate intake, insulin doses, and physical activity levels (steps). Information collected can be presented to patients in a variety of graphical outputs. Despite the availability of several technical means, a large percentage of patients do not reach the goals established in their diabetes treatment.

**Objective:**

The objective of the study was to evaluate the benefits of the Diani telemedicine system for the treatment of patients with type 1 diabetes mellitus.

**Methods:**

Data were collected during a 24-week feasibility study. Patients responded to the World Health Organization Quality of Life – BREF (WHOQOL-BREF) questionnaire and a system evaluation questionnaire. The level of glycated hemoglobin (HbA_1c_) and the patient’s body weight were measured, and the patient’s use of the telemedicine system and their daily physical activity level were monitored. All data were sent from the patient’s device to the Diani server using a real-time diabetes diary app. Wilcoxon and Friedman tests and the linear mixed effects method were used for data analysis.

**Results:**

This study involved 10 patients (men: n=5; women: n=5), with a mean age of 47.7 (SD 19.3) years, a mean duration of diabetes of 10.5 (SD 8.6) years, and a mean HbA_1c_ value of 59.5 (SD 6.7) mmol/mol. The median number of days the patients used the system was 84. After the intervention, the mean HbA_1c_ decreased by 4.35 mmol/mol (*P*=.01). The patients spent 18.6 (SD 6.8) minutes on average using the app daily. After the intervention, the number of patients who measured their blood glucose level at least 3 times a day increased by 30%. The graphical visualization of the monitored parameters, automatic transmission of measured data from the glucometer, compatibility, and interconnection of individual devices when entering data were positively evaluated by patients.

**Conclusions:**

The Diani system was found to be beneficial for patients with type 1 diabetes mellitus in terms of managing their disease. Patients perceived it positively; it strengthened their knowledge of diabetes and their understanding of the influences of the measured values on the management of their disease. Its use had a positive effect on the HbA_1c_ level.

## Introduction

### Diabetes Mellitus

Diabetes mellitus is a major public health problem that is approaching epidemic proportions worldwide [1]. The prevalence of diabetes mellitus is increasing at an alarming rate. The World Health Organization (WHO) reported the global prevalence of diabetes in adults (20 to 79 years) as 8.5% in 2014 [1] and 10.5% in 2021, and the estimated prevalence of diabetes is expected to rise to 12.2% in 2045 [2]. Between 5% and 10% of patients have type 1 diabetes [3]. In 2021, there were about 8.4 million people worldwide with type 1 diabetes. Of these, 83% were aged ≥20 years [4].

The increase in global health expenditure due to diabetes has been considerable, growing from US $232 billion in 2007 to US $966 billion in 2021 for adults aged 20 to 79 years. Direct costs of diabetes are expected to continue to increase. The International Diabetes Federation estimates that total diabetes-related health expenditure will reach US $1.03 trillion by 2030 and US $1.05 trillion by 2045 [5]. Health systems are under pressure related to the sustainability of health care provided at acceptable levels and with a high number of consultations in both primary care and hospitals. Social systems are exposed to increased costs because of the reduced working capacity. As a result of this pressure, patients with diabetes can develop feelings of reduced quality of life [6]. Regular checks on a person with diabetes by a health care provider are generally performed once or twice a year, so diabetes care is primarily based on self-care [6].

The overall average glycated hemoglobin (HbA_1c_) for patients with type 1 diabetes was 66 mmol/mol (average HbA_1c_ values are 58-62 mmol/mol for patients aged 30 years and 58 mmol/mol for patients aged 65 years; data also include insulin pump users) [7].

Currently, different technological tools are available for patients with diabetes mellitus that can significantly facilitate their daily life with diabetes and, at the same time, adapt to their individual needs. Telemedicine systems (TSs) have the potential to offer a solution to the sustainability of health care systems, maintaining or increasing the quality and accessibility of health care and managing large amounts of health-related data with limited resources [8,9].

TSs have been increasingly used in the care of diabetes in the last decade [9-17]. Despite the benefits they offer their users [9,18-20], they have not yet been widely adopted as a tool for health care delivery [10], and the systems have not been adequate enough to meet their created plan for the care of diabetes [5,8,12]. During the recent COVID-19 pandemic, the situation generally improved.

However, the technologies themselves will not help the patients if they are unable or unwilling to fully use what the given technology offers. It is necessary to ensure frequent interaction with the technology. To find the right motivation for a given patient, it is necessary to know the patient’s involvement in terms of treatment, in situations in which it is difficult to self-manage diabetes mellitus, as well as mental abilities and environment [21]. For long-term sustainable TS use by patients with diabetes, the associated expenditure of time, energy, and money must be at a tolerable level in terms of the patient’s treatment burden [22].

Diani is a TS for the management of diabetes mellitus and other chronic diseases, which reports a significant improvement in HbA_1c_ values in patients with type 1 diabetes [21]. The feasibility of an intervention with the Diani TS might fill the knowledge gap in the implementation of TSs in the health care system; explore the reliability, accuracy, and efficiency of the system more closely; and help to identify patient requirements for a digital tool for diabetes care so that such technical solutions lead to their long-term use with a positive impact for all users, patients with diabetes, health care providers, and the national health system.

### Objective of the Study

The main objective of the study was to evaluate the Diani TS for the treatment of patients with type 1 diabetes mellitus, especially its effect on HbA_1c_ and body weight. Specific objectives were to identify the benefits and limitations of the system from the perspective of patients, namely whether the system contributes to a sense of security, whether patients have educational benefits as a result of using the system, whether they feel more confident in managing diabetes care, what functions the system likes to use and which they have not used, and why. The parallel target was to monitor the patient’s views on the operation and use of the system and to evaluate whether the Diani TS affects their health and quality of life. Furthermore, this study aims to determine how patients have used the system (frequency and duration of using particular sections of the Diani TS and access method).

## Methods

### Research Design

The study has been designed as a feasibility study focusing on the evaluation of the Diani TS based on a questionnaire survey of patients with type 1 diabetes mellitus and the use of the Diani TS, which also includes tracking patients’ use of the system. The monitored parameters were HbA_1c_ and the body weight of the patient with diabetes. The study followed the principles of the randomized, crossover intervention study (real-world study) [23,24]. The study design was planned so that the observed parameters of diabetes self-management, involving the use of the Diani TS, are measured in the real conditions of the patient’s daily life.

In a selected internal medicine clinic, which provides comprehensive diagnostic and therapeutic care to both outpatients and hospitalized patients with diabetes mellitus from all over the Czech Republic, 10 patients were recruited and included in this study, with an emphasis on maintaining the highest representativeness.

#### Diani

**Figure 1:**
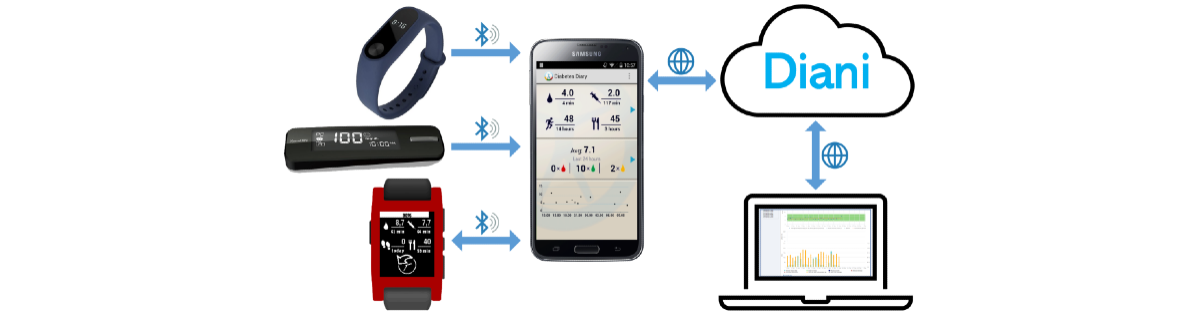
The Diani telemedicine system.

The Diani TS configuration used in the study consisted of a web app (Diani), a mobile app called the Diabetesdagboka (DDB) diabetes diary (installed on the Samsung Galaxy J5 Android 8.1), a Fora Diamond MINI Bluetooth glucometer (FORA Care Suisse AG), and a Fitbit Flex smart bracelet (Fitbit Inc). A continuous blood glucose monitor (xDrip module, Nightscout) and a smartwatch (Pebble Classic), with the DDB app for the Pebble Smartwatch, were all connected to the system, allowing for an alternative way of entering information into the DDB app (Figure 1). The monitored data were downloaded from the device or automatically sent to a web application, where they were automatically sorted, aggregated, analyzed, and saved [21,25,26].

### Study Outcomes

#### Primary Outcomes

HbA_1c_ values and patient body weight were determined at each of the 3 physician visits during the study (visits 1-3, as described below). The parameters were collected in a diabetological office, including taking a blood sample, which was evaluated by the internal hospital laboratory. The primary objective was to check whether there was a significant change in these values after the intervention. The secondary objective was to see if there was a change in these values at V3 (ie, at the end of the study).

#### Comparison With Prior Work

A growing number of studies discuss the influence of digital technologies on the compensation of diabetes mellitus disease [9,18-20,27]. Interventions are more often in the form of the use of mobile apps, web portals, email, SMS text messages, phone calls, and customized smart devices [6,9,27]. Interventions can include diabetes education, nutritional interventions, a physical activity plan, and blood pressure management [9,28,29]. Some technologies allow real-time data sharing, such as glucometers [29,30]. Feasibility studies include semistructured interviews or questionnaires focusing on the quality of life [9] and the usability and acceptability of the technical solution [6,9] in a standardized form [31] or created by the authors [6,27]. Technical equipment and consumables were provided to participants in some studies [29]. The study varies in scope in the number of patients (10 to 2378) and duration of 3 to 12 months [9]. A common problem is patient recruitment and a high patient dropout rate [9]. Telemedicine may be a useful supplement to usual clinical care to control HbA_1c_, at least in the short term, but there was no evidence of a convincing effect on the quality of life [9]. Telemedicine interventions appeared to be most effective when they use a more interactive format [9].

### Secondary Outcomes

To determine the characteristics of the group, patients completed the patient questionnaire at the initial visit (V1). To determine the possible impact of the Diani TS on diabetes self-management, selected questionnaire questions were submitted for completion at subsequent visits (V2 and V3). To study the impact of the Diani TS on the quality of life of patients, patients completed a standardized WHO questionnaire at each visit (V1, V2, and V3).

Another partial objective was to find the frequency of using particular Diani TS features and to register the time needed to enter and collect data. For this, a Matomo analytics application was used with a focus on tracking the patient’s use of the Diani system at the time of the intervention. To evaluate the Diani TS and identify the strengths and weaknesses of the system, which is important for its further innovation, patients completed a product questionnaire (described in the *Key Instruments* section) during a postintervention visit.

### Inclusion and Exclusion Criteria

The study included patients with type 1 diabetes mellitus, who were ≥18 years old, and who administered insulin subcutaneously by means of an insulin pen, using a personal glucometer, a smartwatch, and a smartphone. Exclusion criteria were pregnancy, insulin pump therapy, inability to use the Diani TS, and engagement with the study criteria.

During V1, eligibility and exclusion criteria were considered and assessed based on medical records, patient interviews, and laboratory tests.

### Study Sample

The physician offered to participate in the study for all consecutive patients with type 1 diabetes treated with insulin for at least 1 year until the quota was reached.

The study involved 10 patients (5 women and 5 men). The mean age of the participants was 47.7 (SD 19.3) years; the mean duration of diabetes was 10.5 (SD 8.6) years; and the mean BMI was 26.9 (SD 3.6). The mean body weight of the patient at the time of study entry was 81.1 (SD 16.9) kg and the mean HbA_1c_ was 59.5 (SD 6.7) mmol/mol. The median number of days for which patients tested the system was 84.

### Data Collection

**Figure 2:**
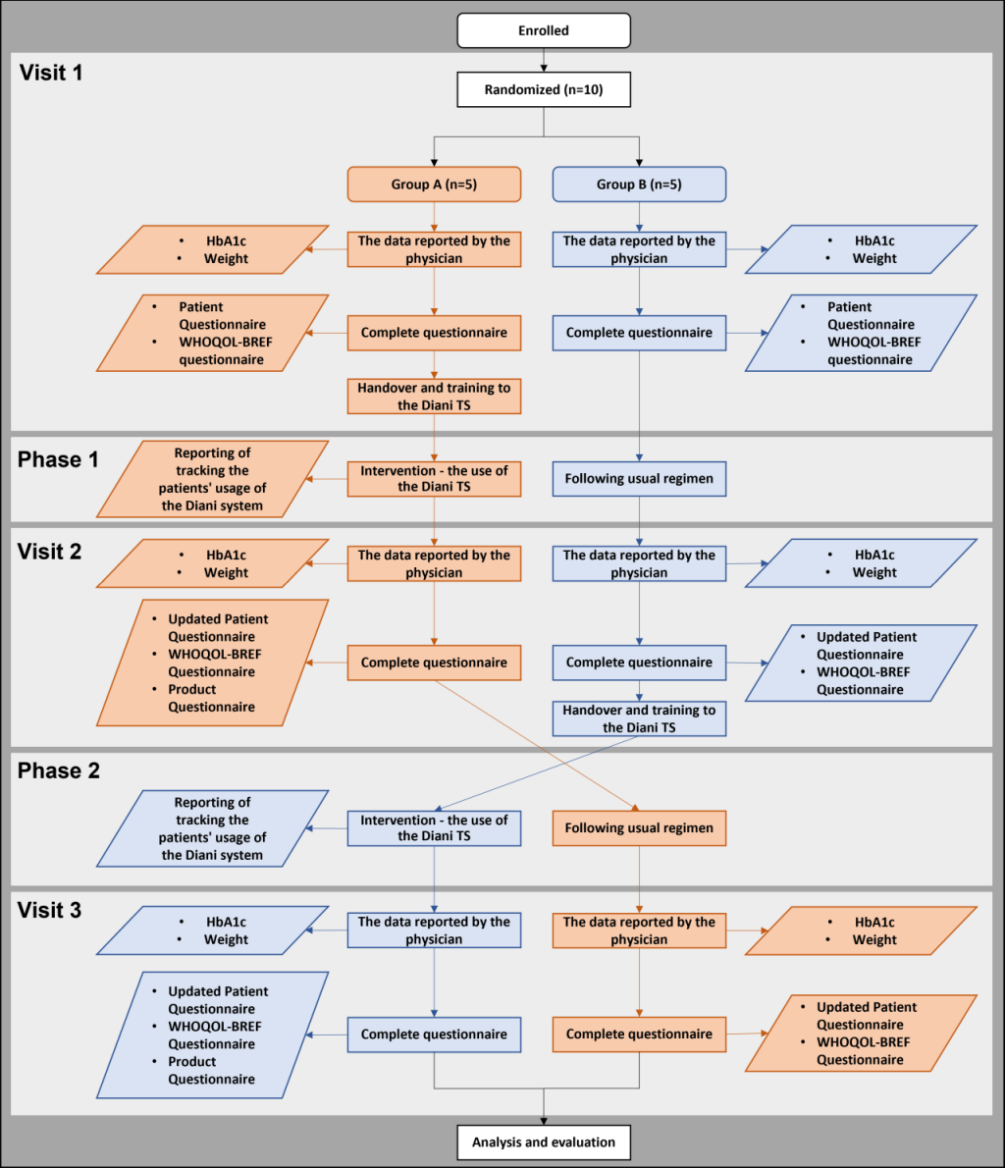
Study scheme (group A: orange; group B: blue). HbA_1c_: glycated hemoglobin; TS: telemedicine system; WHOQOL-BREF: World Health Organization Quality of Life – BREF.

Data for the Diani TS evaluation were obtained within the feasibility study, which had the characteristics of a randomized crossover intervention study. The total duration of the study was 24 weeks (Figure 2). The patients were randomly divided into 2 groups: group A and group B. Patients in group A used the system for the first 12 weeks; patients in group B followed their usual plan of care for diabetes. After 12 weeks, the patient’s activity changed, the patients in group A followed their usual plan of diabetes care, and the patients in group B used the Diani TS for self-management of diabetes again for 12 weeks.

The study included 3 visits to the doctor: the initial visit (V1), then a follow-up visit (V2) after 12 weeks of study, and the third visit (V3) after 24 weeks of study.

#### Enrollment Visit (V1)

During V1, the physician evaluated the inclusion and exclusion criteria. The physician asked the patient if they were willing to participate in the project and asked them to sign an informed consent. The patients enrolled in the study received a set of questionnaires (the patient questionnaire, the World Health Organization Quality of Life – BREF [WHOQOL-BREF] questionnaire), which they completed during V1. If the patient was assigned to group A, a Diani TS monitoring kit was given and they were trained in the use of the system. The patients in group A were supposed to use the Diani TS between V1 and V2, optimally for 12 weeks. The data reported by the physician were obtained from medical records and recorded in the Diani web app. The data included HbA_1c_ and the patient’s body weight.

#### Follow-up Visits (V2 and V3)

V2 and V3 took place within the usual practice in the clinic at 3 and 6 months after V1. The period between weeks 1 and 12 was referred to as phase 1 and the period between weeks 13 and 24 was designated as phase 2. During V2 and V3, the patient completed an updated patient questionnaire (questions from the Demographic section were omitted) and the WHOQOL-BREF Questionnaire. At the end of the intervention, patients completed the product questionnaire (group A at V2 and group B at V3). At V2, patients in group B received the Diani TS monitoring kit and were trained in how to use the system. Patients in group B used the Diani TS between V2 and V3 (weeks 12-24); patients in group A followed their usual plan of diabetes care during this time, as before the Diani TS intervention (without further intervention). The data reported by the physician were obtained from medical records and recorded in the Diani web application. The data collected were HbA_1c_ and the patient’s body weight.

### Key Instruments

#### The Patient Questionnaire

The patient questionnaire was developed by the research team involved in this particular study to obtain data describing important areas related to insulin therapy and diabetes in general. It focuses on the details of the patients and their technical skills in using conventional computing and smartphones in order to assess their ability to use the Diani TS. The questionnaire consists of 43 questions classified into the following categories: demographic data; self-management of diabetes and health, including patient’s eating habits of the patient; physical activity; questions that determine the level of use of computer technology and smartphones.

#### The WHOQOL-BREF Questionnaire

The WHOQOL-BREF Questionnaire is a standardized questionnaire by the WHO. The questionnaire focuses on determining the quality of life of the patient. It is capable of detecting the current deterioration of health and capturing differences in the living conditions of people with long-term illness or disability [32,33]. The questionnaire consists of 26 questions. Twenty-four questions represent four areas, which are as follows: (1) physical health, (2) psychological area, (3) social area, and (4) environment. Two questions are evaluated separately; they deal with the evaluation of quality of life and overall health. Items are evaluated on a 5-point Likert scale.

#### The Product Questionnaire

The product questionnaire is a questionnaire created by this study’s research team and focuses on the evaluation of the Diani TS by an intervention group. It is based on standard product evaluation research [34-37] and is adapted for study purposes. Due to the simplicity of the questions, the questionnaire was not validated. The questionnaire contains 18 questions that assess patients’ experience with the use of the Diani TS, of which 5 questions were open for verbal evaluation of the system, describing both positive and negative characteristics of the system and possible suggestions for improvement.

#### Tracking the Usage of the Diani TS by the Patient

To track the patients’ usage of the Diani TS, the open-source analytic framework for web analytics, Matomo, was used. This software monitors the digital visits of patients to the Diani TS and shows reports for further analysis. The objective was to identify how often individual users log into the system, how long they stay there, what sections of the system they use or what activities they perform (value registration, data viewing, etc), and in what way (access to individual features).

### Study Interventions

The intervention consisted of using the Diani TS by patients to self-manage diabetes for a predetermined period of time. Patients had access to the Diani web app, the DDB mobile diabetes diary app, the Fitbit smart bracelet, the Pebble smartwatch with the DDB Companion for the Pebble smartwatch app, and the Diamond Mini glucometer.

### Statistical Analysis Plan

The primary objective was to identify a potential change in HbA_1c_ and the patient’s body weight after the intervention and at the end of the study (V3), at the selected significance level of .05. Another objective was, at the same level of significance, to identify a potential change in the patient’s quality of life after the intervention. For this purpose, a regression analysis, the linear mixed effects (LME) method, and nonparametric tests (Wilcoxon and Friedman tests) were performed.

The secondary objective was to determine how frequently the patients used the Diani TS features and when they used them. As with the analysis of the characteristics of the group, the basic descriptive statistical analysis of the primary data (mean, SD, median, upper and lower quartiles, and minimum and maximum rates) was used here as well.

For nonstandardized questions, a list of individual responses, their categorization, and coding into numerical values were performed. For the free text questions, a thematic evaluation of the answers was performed.

### Ethics Approval

All procedures were performed according to the Ethics Committee for Multicenter Clinical Trials of Motol University Hospital and the 2nd Faculty of Medicine, Charles University in Prague, Czech Republic (approval date: November 4, 2016). All participants signed an informed consent form.

## Results

### Diabetes Self-management and Health

All patients agreed that they had been instructed on lifestyle changes and diabetes care during their treatment. Seven (70%) patients checked their blood glucose values at least 3 times a day before entering the study, and 10 (100%) patients checked their blood glucose at least 3 times a day after the intervention. Of these, 6 (60%) participants tested their blood glucose at least 4 times a day, which was 20% more than at the beginning of the study (Table 1). Using the LME method, it was found that during the Diani system use period, patients measured their blood glucose on average 1.8 times more often (*P*=.06).

When asked during V1, 4 (40%) patients said that they suffered from depression. This number of cases may seem high, the likely reason being that the term depression was not specified or defined in the questionnaire and patients could thus explain it in different forms. Three (30%) respondents did not feel like managing their lives due to type 1 diabetes mellitus. Seven (70%) patients said that the behavior change plan would help them better compensate for type 1 diabetes mellitus; 6 (60%) patients would like more information in the field of nutritional therapy and information about type 1 diabetes mellitus disease.

Seven (70%) patients reported that they could reduce the risk of health complications resulting from diabetes mellitus. Seven out of 10 participants stated that they had a diet plan, but only 2 participants always followed it and 4 occasionally, while the others only rarely or never. Nine (90%) patients stated that they had bigger problems counting carbohydrates and 4 (40%) of them did not manage this activity at all. At the same time, 6 (60%) patients reported eating the recommended portion sizes of food almost always. Half of the participants felt that it was difficult for them to manage their illness and all of them stated that they were motivated to work on their treatment.

**Table 1:** Comparison of outcomes before and after the intervention (N=10).

Intervention				Before		After
**Diabetes self-management**						
	**Blood glucose measurement frequency, n**					
		1× per day	2		0	
		2× per day	1		0	
		3× per day	3		4	
		4× per day	3		5	
		>4× per day	1		1	
**Targeted physical activity**						
	**Type of physical activity,^a^ n**					
		Cycling	5		4	
		Walking	9		8	
		Physical work (in the garden)	7		3	
		Housework	1		0	
		Running	2		1	
		Exercise (gym)	2		2	
		Dance	1		1	
	**Length of physical activity, n**					
		I do not perform physical activity	1		0	
		1-29 min	0		0	
		30-59 min	5		6	
		60-119 min	1		2	
		≥120 min	3		2	

^a^Multiple answers could be chosen for the question.

### Physical Activity

At V1, 9 (90%) patients reported targeted, planned, and structured physical activity at least once a week, of which 6 (60%) reported, more than twice a week, mostly walking, gardening, or cycling. This activity usually lasted 30-60 minutes. After the intervention, the type of activities decreased (Table 1), but the duration of the activity increased slightly, and the frequency remained the same. Three out of 10 participants felt their physical condition was deteriorating; after the intervention, 4 out of 10 patients had the same feeling.

### Technical Experience

Nine (90%) of the patients had no experience with using any mobile diabetes diary application prior to participating in this particular study. One patient stated that he had experience with the DDB mobile app. None of the study participants had participated in any study that dealt with diabetes treatment assistance before entering our study.

### The WHOQOL-BREF Questionnaire

The return rate of the questionnaires was 80%. For the first separate question on the quality-of-life assessment, 60% (n=6) of the participants rated their quality of life as good or very good at V1. At V2 and V3, 78% (n=7) and 89% (n=8) of the participants chose these answers, respectively. The second separate question focused on the evaluation of satisfaction with the participants’ health. At V1, 30% (n=3) of the patients expressed satisfaction with their health; 44% (n=4) and 50% (n=5) of the participants were satisfied with their health at V2 and V3, respectively.

The Friedman test did not show significant differences in quality of life within the group between individual study phases. The Wilcoxon test did not show a change regarding the intervention or nonintervention groups.

### The System Evaluation Questionnaire

As part of the system evaluation, all users marked the use of the system as socially discreet. Its use (data entry, daily use, and charging) was rated by 90% (n=9) of the patients as rather easy or easy. Similarly, 90% (n=9) of users reported spending more time thinking about diabetes mellitus as a result of using the system and managing to dose insulin better. For example, based on links to educational resources from the DDB app, there was new information for one of the patients that their insulin bolus could be adjusted by more than 2 units. Seven (70%) users feel safer as a result of using the system. In terms of time, 70% (n=7) of the patients evaluated working with the system to be rather undemanding or not at all demanding.

#### Patient-Reported Positive Aspects of the System

In open questions, users positively evaluated the graphical visualization of measured parameters (clarity of monitoring results and digital form), automatic transmission of measured data from the glucometer, and compatibility and interconnection of individual devices when entering data. One of the system benefits was also reported to be the simplicity of entering data into the mobile app and the generally simple operation of the system. The possibility of connecting a continuous blood glucose monitor and the smart bracelet, digitizing data, and a possible view from several types of devices (interconnection of the system with the internet) was appreciated. The chronological presentation of the key indicators was also positively assessed. These indicators enable the evaluation, comparison, and search for connections between carbohydrate intake, physical activity, and the size of the insulin dose. Patients acknowledged the opportunity to print the monitored values with the selected data filter and decide the amount of data, for easier perception of the information. In the Diani TS evaluation, users emphasized the power of the system in providing feedback to the user, which makes it easier for them to understand the measured values and information in context and as a result of their behavior, to improve diabetes management in the future.

#### Patient-Reported Negative Aspects of the System

The provided smartwatches were reported to be difficult to control (small and rigid buttons) and negatively evaluated. Users also requested the display of key values in a single graph and the depiction of trends in the development of the measured values (curve fitting). Furthermore, the need to link the Diani TS with Kalorické Tabulky (a Czech food composition database mobile app) was requested, and a requirement to automatically add up the registered values of the received carbohydrate intake over a predefined period was mentioned. Users also described the acquisition and registration of data as time-consuming. Another issue was the overuse of devices (both a smartwatch and a smart bracelet placed on the wrist). During the study, patients reported communication issues with the server. This outage was also described as unsatisfactory. This was mainly due to a poor internet connection on the users’ mobile phone, Bluetooth being turned off in the device, or improperly timed technical maintenance of the server. However, these technical issues were eliminated during the study. During these episodes, all data were stored in the user’s mobile phone and were uploaded to the Diani server after the connection was re-established, which occurred within 5-15 minutes.

#### Patients’ Suggestions for Improving Diani

Suggestions for improving the system included the possibility of adjusting the limit values of the monitored blood sugar level. The need for retrospective control of data transfer (in the form of notification) and access to the application for devices operating on the iOS operating system were also wanted features. Additionally, the need to improve the smartwatch so that it is more user-friendly was also stated. Furthermore, a proposal to reduce a large number of items was mentioned in the web version of the application and to emphasize the monitoring of exceeding carbohydrate intakes was mentioned.

Five (50%) users expressed their readiness to pay for the use of the system; 3 (30%) users are not willing to pay for the system; and 2 (20%) are unable to decide. One (10%) user would be willing to spend 5%-10% of their monthly income on the system; 40% (n=4) users would be willing to spend between 1% and 5% of their monthly income; and 20% (n=2) user would be willing to spend less than 1% of their monthly income (n=2, 20% of the participants reached the average net wage in the Czech Republic [the median net wage for 2017 in the Czech Republic was CZK 25,021; US $1070]; the others had a lower wage or did not answer the question).

### HbA_1c_ Level and Body Weight Changes

At V1, the mean HbA_1c_ level was 59.5 (SD 6.7) mmol/mol; after the intervention, the mean HbA_1c_ decreased by −4.35 mmol/mol (*P*=.01) (LME analysis). The effect comparing monitoring at V2 with V1 was −6.54 mmol/mol (*P*=.04); the effect comparing the use of the Diani TS at V3 with V1 was −2.16 mmol/mol (*P*=.45). The mean HbA_1c_ at V2 was 60 (SD 9.8) mmol/mol and at V3 was 61.8 (SD 6.1) mmol/mol in participants who participated in the intervention in phase 1.

The average body weight of participants was 81.4 (SD 16.4) kg at V1, 81.1 (SD 16.9) kg after intervention, and 81.5 (SD 16.9) kg at V3. Statistical analysis did not show a significant difference (*P*>.05) in patients’ body weight between the intervention or control groups; there was even no effect within one group during the study.

### Patient Usage of the Diani System

For the duration of the study, the patients spent 41.6 (SD 31.7) hours on average using the DDB app, with a daily average use of 29.7 (SD 22.7) minutes. Of the total time, the daily average of 11.6 (SD 18.6, 67%) minutes was spent on manual physical activity registration, 3.5 (SD 4.6, 17%) minutes on the registered blood glucose level, 2.1 (SD 2.9, 9%) minutes on carbohydrate registration and 1.4 (SD 1.2, 7%) minutes on insulin dose registration. The remaining time includes inactive use of the application—viewing records, launching the application on the smartphone, and communicating with the server. On average, patients visited the DDB application 4.5 (SD 2.3) times a day and performed 31.3 (SD 23.9) activities per day; these activities include: registering items, viewing records, and editing data in the application. The average number of activities per visit was 7.1 (SD 2.9), and 29.4% (SD 13.3%) of the visits left the application immediately after viewing an item. The length of 1 visit lasted 14.2 (SD 11.9) minutes on average. As for the length of app use, the average time of the visit decreased during the intervention. Two users had a minimum number of entries in the DDB and were excluded from the objective evaluation of the system due to misrepresentation of results. A comparison of the average values of the daily number of visits and activities performed per day showed that one-third of patients used the system very actively and, conversely, 20% (n=2) of users used the system below average. Seven (70%) patients with a higher degree of involvement with the Diani TS actively used the system even a year after the study ended.

## Discussion

### Overview

This study was the first clinical trial to evaluate the Diani TS for users with type 1 diabetes mellitus. On the basis of the data obtained, recommendations were subsequently derived, and suggestions for further innovation of the Diani TS were determined.

### Principal Results

The most significant result of the evaluation was the decrease in HbA_1c_ level associated with participating in the study and the increase in the frequency of blood glucose measurements during self-management control after using the Diani TS. This increase was observed in 50% of the participants and is interpreted as a positive effect of the system. The reason can be that the system allows the patient both to observe the data more and to display it in the context of activities and might motivate patients to improve the measured data.

Data from the patient questionnaire showed that determining the correct content of carbohydrates in the diet is a major challenge. Most (90%) of the respondents admitted that they had this problem; nevertheless, 60% of patients also stated that they consumed the recommended amount of carbohydrates in their diet. The focus on diet should be approached more actively. In the future, it would be interesting to focus on this topic and think about how to motivate patients to learn from the data they enter into the system and how to educate them to use the data and explain the importance of a well-balanced diet plan to compensate for the challenges of diabetes mellitus. This problem was the reason for the integration of the food composition mobile app Kalorické Tabulky [38] directly into the Diani TS.

Regarding the quality-of-life assessment (WHOQOL-BREF questionnaire), the trend curve indicated an increase in the perception of one’s quality of life and health. Unfortunately, due to the low return rate of the questionnaires (80%) at the end of the first and second phases of the study, the results cannot be considered entirely reliable.

In general, the Diani TS was positively evaluated, in terms of graphics, functionality, and practical use. The system was perceived as user-friendly, well organized, and motivating by most of the participants. The participants appreciated the feedback provided by the Diani TS, especially concerning the complexity and orderliness of the monitored data submitted in real time. Based on comprehensive information, patients reported that they were able to perceive and understand the relationship between particular monitored values (physical activity, carbohydrate intake, and insulin bolus size), which allowed them to better estimate insulin dose. The system enabled patients to deepen their knowledge in their current understanding of blood glucose variations, in general. As a result of the use of the Diani TS, the patients stated that they felt safer and that it was easier for them to leave their comfort zone, for example, when traveling and sleeping outside their place of residence. Eliminating stressful situations like these can have a positive effect on the patient’s quality of life.

The accessories of the system, namely the smartwatch, the use of which did not bring patients a large benefit compared to the efforts made to become familiar with them, were negatively evaluated. Proposals for additional innovations to the system were identified on the basis of subjective evaluation.

Based on the system logs, the operation of the system was not very time-consuming for patients. They did not spend more than 18.6 (SD 6.8) minutes a day registering the data, which was why the Diani TS was perceived as easy to use and the patients evaluated its use as discreet and not interfering with daily activities.

### Limitations

During the study, there were some technical issues regarding the transfer of information from the glucometer to the Diani server. These issues were resolved during the first phase of the study. In case the problems were caused by users’ lack of knowledge of the system, assistance for solving respective problems was provided by means of a telephone conversation or an electronic conversation. More detailed instructions on using the system or video instructions may be appropriate in the future.

Difficulties in recruiting participants have been described in previous studies [39], and measures were therefore taken to reduce problems with recruiting participants for the study by minimizing inclusion criteria. Patients were strongly motivated to participate and remain in the study so that they could receive free glucose strips for glucometers and lancets; there was also the possibility of keeping the test set (glucometer, mobile phone, and smart bracelet) after the end of the study. Another important factor was the availability of new technologies and information in the field of diabetology. Still, there appeared difficulties in recruiting patients for the study, as only 10 of the 30 planned patients were recruited.

### Comparison With Prior Work

Significant effects on the reduction of HbA_1c_ were confirmed among patients who used the Diani TS. The system also helped strengthen the patient’s position in terms of a positive effect on compensating for type 1 diabetes mellitus. Patients expressed a generally positive perception of the system.

Similar studies have been conducted, but they differ in their study design, patient variability, and technologies used. These studies have similar results in terms of a positive effect on HbA_1c_ [6,9,40,41] and an unproven effect on the quality of life [9]; however, the results cannot be compared closely due to the diversity in data collection and evaluated information.

### Conclusions

The feasibility study was a valuable first step in the evaluation of the Diani TS. It helped identify both the benefits and limitations of the system from the users’ perspective, recorded their views on the operation and use of the system, and assessed whether the Diani TS had an impact on their health and quality of life. Based on the information obtained, new needs and demands of patients for the TS were identified, from which recommendations were derived, and suggestions for further innovation of the Diani system were determined. At the same time, the study was helpful as a real test of the system features. In the future, the methodological findings of the study will be used in the evaluation of the Diani TS extended with other components and functions. The study confirmed both that the Diani TS can be beneficial for patients with type 1 diabetes mellitus in managing their disease and that its use can (at least in the short term) increase interest and motivation to follow medical recommendations. The effectiveness of the TS for managing diabetes is limited by its acceptance of patients. We need more information about what motivates the long-term use of the system by patients with diabetes as well as the degree of influence imposed by personality.

## Abbreviations

DDB: Diabetesdagboka
HbA_1c_: glycated hemoglobin
LME: linear mixed effects
TS: telemedicine system
WHO: World Health Organization
WHOQOL-BREF: World Health Organization Quality of Life – BREF
